# Transcriptome Analysis of Long Non-Coding RNA in the Bovine Mammary Gland Following Dietary Supplementation with Linseed Oil and Safflower Oil

**DOI:** 10.3390/ijms19113610

**Published:** 2018-11-15

**Authors:** Eveline M. Ibeagha-Awemu, Ran Li, Pier-Luc Dudemaine, Duy N. Do, Nathalie Bissonnette

**Affiliations:** 1 Agriculture and Agri-Food Canada, Sherbrooke Research and Development Centre, Sherbrooke, QC J1M 0C8, Canada; ran.li1986@hotmail.com (R.L.); pier-luc.dudemaine@canada.ca (P.-L.D.); ngoc.d.do@mail.mcgill.ca (D.N.D.); nathalie.bissonnette@canada.ca (N.B.); 2 College of Animal Science and Technology, Northwest A&F University, Yangling 712100, China; 3 Department of Animal Science, McGill University, Ste-Anne-De-Bellevue, QC H9X 3V9, Canada

**Keywords:** long non-coding RNA, bovine mammary gland, linseed oil, safflower oil, lipid metabolism, fatty acid synthesis, *cis*-regulation

## Abstract

This study aimed to characterize the long non-coding RNA (lncRNA) expression in the bovine mammary gland and to infer their functions in dietary response to 5% linseed oil (LSO) or 5% safflower oil (SFO). Twelve cows (six per treatment) in mid lactation were fed a control diet for 28 days followed by a treatment period (control diet supplemented with 5% LSO or 5% SFO) of 28 days. Mammary gland biopsies were collected from each animal on day-14 (D-14, control period), D+7 (early treatment period) and D+28 (late treatment period) and were subjected to RNA-Sequencing and subsequent bioinformatics analyses. Functional enrichment of lncRNA was performed via potential *cis* regulated target genes located within 50 kb flanking regions of lncRNAs and having expression correlation of >0.7 with mRNAs. A total of 4955 lncRNAs (325 known and 4630 novel) were identified which potentially *cis* targeted 59 and 494 genes in LSO and SFO treatments, respectively. Enrichments of *cis* target genes of lncRNAs indicated potential roles of lncRNAs in immune function, nucleic acid metabolism and cell membrane organization processes as well as involvement in Notch, cAMP and TGF-β signaling pathways. Thirty-two and 21 lncRNAs were differentially expressed (DE) in LSO and SFO treatments, respectively. Six genes (*KCNF1*, *STARD13*, *BCL6*, *NXPE2*, *HHIPL2* and *MMD*) were identified as potential *cis* target genes of six DE lncRNAs. In conclusion, this study has identified lncRNAs with potential roles in mammary gland functions and potential candidate genes and pathways via which lncRNAs might function in response to LSO and SFA.

## 1. Introduction

Advances in high throughput RNA sequencing technologies and computational prediction techniques have enabled the discovery of an abundant class of non-coding RNA (ncRNA) species with emerging roles in gene regulation. Among these, long non-coding RNA (lncRNA) generally considered as RNA molecules >200 nucleotides (nts) are known to participate in a diverse set of biological processes including genomic imprinting, X chromosome inactivation, cell differentiation and development, cancer metastasis, immunity, disease and ageing [1,2,3,4,5,6,7,8,9]. LncRNA mediate these processes through diverse mechanisms including acting as scaffolds, decoys or signals, regulation of gene expression in *cis* or *trans* and antisense interference or by epigenetic regulation, organization of protein complexes, cell-cell signaling, allosteric regulation of proteins as well as genome targeting [7,10,11,12].

To date, a large number of lncRNA genes, enabled by continued developments in high-throughput sequencing methodologies, have been identified in the genomes of human (*n* = 96,308), mouse (*n* = 87,774), cow (*n* = 22,227), rat (*n* = 22,217), gorilla (*n* = 15,095), other animals and model organisms (http://www.bioinfo.org/noncode/analysis.php, accessed on 03 April 2018). Although the function of majority of lncRNAs are unknown, the mode of action of a few like X inactive specific transcript (XIST, functions in X chromosome inactivation, chromatin modification etc.) [7,13,14], HOX transcript antisense RNA (HOTAIR, functions in positional identity, regulate gene expression in *trans* and is associated with a variety of cancers) [15,16,17] and metastasis associated lung adenocarcinoma transcript 1 (*MALAT1*, functions in nuclear structure organization and is associated with a variety of cancers etc.) [18] are well characterized. In bovine, only a few studies have examined the occurrence of lncRNAs in muscle [19], skin [20], expressed sequence tag (EST) data [21,22], across 18 tissues [23] and in the mammary gland [24]. Although it has been predicted that bovine ncRNAs including lncRNAs are abundant, primarily intergenic and associated with regulatory genes [22], little is known about the functions of lncRNAs in the bovine genome and the lncRNA atlas of the different cell types and tissues remain to be explored. A recent study has suggested that some lncRNAs play a role in translation control of target mRNA (messenger RNA) during development of bovine early embryos [4] as well as development processes in calf gut at the early part of life [25].

Numerous studies in humans and mice have shown evidence of a role for lncRNA in mammary development and disease [26]. Pregnancy-induced non coding RNA (PINC) is the first lncRNA shown to be differentially expressed in the mammary gland of a pregnancy simulated rat model [27]. Further work showed that the expression of PINC is temporally and spatially controlled in response to developmental stimuli in vivo and loss-of-function analysis suggest roles in cell survival and regulation of cell-cycle progression in the mammary gland [28]. Zfas1 also known as ZNFX1 antisense RNA 1 is a lncRNA localized in the ducts and alveoli of the mammary gland whose expression is differentially regulated during different stages of pregnancy, lactation and involution [29]. Furthermore, knockdown of *Zfas1* in a mammary epithelial cell line (HC11 cells) promoted increased cellular proliferation and differentiation and thus is a key player in the regulation of mammary alveolar development and epithelial cell differentiation [29]. Unlike lncRNA, more efforts have been directed at characterizing microRNA (miRNA, another class of ncRNA) expression and potential regulatory roles in the bovine mammary gland [30,31,32,33,34,35,36,37]. However, the occurrence and roles of lncRNAs in the bovine mammary gland is largely unknown and remain to be explored. Recently, Tong et al. [24] identified 184 lncRNAs (intergenic) in the bovine mammary gland including 36 lincRNAs co-located with 172 milk related quantitative trait loci (QTL) and one lncRNA co-located within a mastitis QTL region. Moreover, lncRNAs have been shown to play roles in dietary response in different species including human [38,39,40], mouse [41], pig [42] and calf [43]. LncRNA roles in dietary responses might be through various processes such as metabolic control [40], glucose homeostasis [40] or hypoxia-mediated metastasis [44]. Recently, Weikard et al. [43] identified 270 differentially expressed lncRNAs in the jejunum mucosa of calves fed two different milk diets and suggested that the lncRNAs might function by modulating biological processes related to energy metabolism pathways and cellular signaling processes influencing the intestinal epithelial cell barrier function. 

It is well documented that bovine milk fat contains isomers (e.g., conjugated linoleic acid (CLA)) that positively influence human health [45,46]. Furthermore, bovine milk fat can be modified to increase the contents of its beneficial components [46]. Particularly, many studies have shown that unsaturated fatty acids enriched dietary supplementation with plant oils (e.g., linseed oil, corn oil, canola oil, safflower oil) and fish oil significantly increased the concentrations of milk beneficial fatty acids such as CLA [47,48,49,50,51]. Previously, we identified numerous differentially expressed genes and miRNAs in mammary gland tissues of cows following dietary supplementation with unsaturated fatty acids enriched diets [32,52,53]. The functions of lncRNAs in this dietary response are not known. In order to shed more light on lncRNA occurrence in the bovine genome, we characterized the lncRNA expression in the bovine mammary gland and examined its expression pattern in response to diets rich in unsaturated fatty acids. Moreover, we also performed lncRNA function enrichment via their potential *cis* regulated target genes.

## 2. Results

### 2.1. Expressed LncRNAs in the Bovine Mammary Gland

One hundred base pairs paired-end RNA sequencing of 36 libraries generated a total of 1.2 billion reads. About 87.2% of reads mapped to unique/multiple positions on the bovine genome UMD3.1 built. Of these, 96.5% mapped to unique positions and were further processed while reads that mapped to multiple positions (3.2%) and discordant alignments (0.38%) were discarded. A total of 27,967 potential transcripts were identified. Since lncRNA expression is generally low as compared to mRNA, only lncRNAs with DESeq2 normalized counts ≥5 and present in at least 10% of our libraries were considered as truly expressed and also used in DE analysis. Consequently, 72.29% (20,218) of potential lncRNA transcripts failed this screening step and were not further considered.

**Figure 1:**
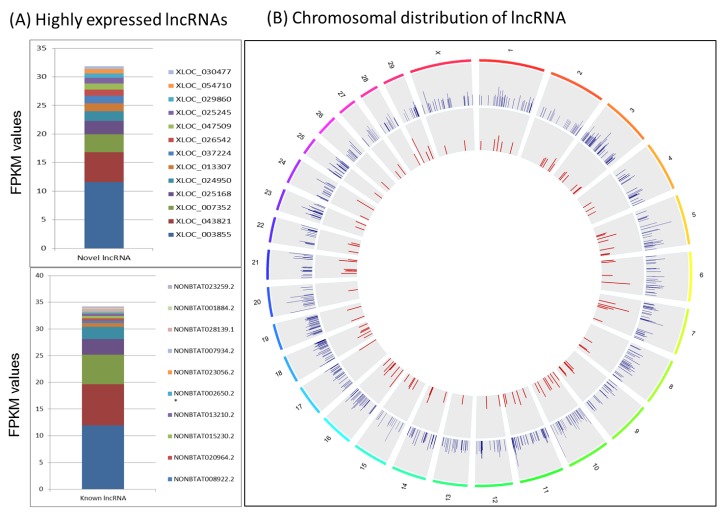
(**A**) Fifteen known and 13 novel highly expressed lncRNAs in the bovine mammary gland. FPKM values ranged from 0.55 to 11.56 or 0.21 to 11.93 for novel and known highly expressed lncRNAs, respectively. (**B**) Intuitive map of lncRNA distribution across bovine chromosomes (outermost circle, different colors). The inner circle (blue lines) represents novel lncRNAs and the innermost circle (red lines) represents known lncRNAs. The height of the line is proportional to the expression level (FPKM) and only those with FPKM > 0.02 are shown.

A total of 4955 lncRNA genes (7749 lncRNA transcripts) equivalent to 325 known and 4630 novel lncRNA genes were identified (Supplementary file 1). Using FPKM (fragments per kilo base of transcript per million mapped reads) normalization, 13 novel and 15 known lncRNAs were highly expressed (0.55 to 11.56 FPKM values for novel or 0.21 to 11.93 FPKM values for known lncRNAs) in the bovine mammary gland (Figure 1A).

The most highly expressed known lncRNAs, NONBTAT026075.2 (FPKM = 11.93) and NONBTAT026069.2 (FPKM = 7.69) are located on the mitochondria (Mt) DNA. The highest number of novel lncRNAs are located on bovine chromosome (BTA) 3, 5, 7,8, 10, 18, 19 and X (209 to 258 lncRNAs) and known lncRNAs on BTA 3 and 10 (20 each) (Figure 1B, Supplementary file 2).

**Figure 2:**
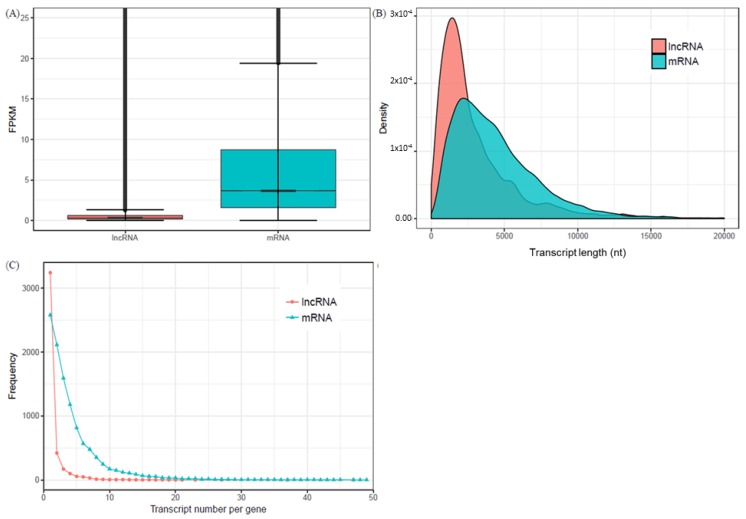
Features of identified lncRNA transcripts compared with transcripts of protein-coding genes. (**A**) Mean expression level of protein-coding mRNA transcripts is 3.6 compared to 0.30 for lncRNA. (**B**) LncRNA transcript length distribution compared with protein-coding mRNA. (**C**) Transcript number per lncRNA gene compared with protein-coding mRNA.

Expression level is a feature that distinguishes lncRNAs from mRNAs. Using FPKM normalization, we showed that the mean expression level of mRNA transcripts from the same data was 3.6 as compared to 0.30 for lncRNA (Figure 2A).

### 2.2. Characteristics of Expressed LncRNAs

LncRNAs are generally regarded as RNA molecules >200 nts. The length distribution of identified lncRNA transcripts ranged from 200 to over 10,000 nts (Figure 2B, Supplementary file 3a). The majority (45.11%) were between 200 and 999 nts followed by 1000 to 2499 nts (37.54%) while 17.34% were ≥2500 nts long. One known lncRNA was however <200 nts long. Compared with mRNA transcripts from the same data [53], transcript length of majority of mRNA was between 500 and 7000 nts (Figure 2B). 

The genomic location of a lncRNA is important as it may give clues to its functions. Thus, identified lncRNAs were classified according to their genomic location and expression direction into 11 classes (Supplementary file 3c). As expected, 62.38% of lncRNA transcripts were intergenic and located at >1 kb (kilo base pairs) away from the nearest gene. This was followed by an appreciable number (23.86%) of transcripts located in such a way that one or more of their exons overlapped with the exons of protein coding genes. LncRNA transcripts located within a one kb region upstream of protein coding genes and transcribed in the same or opposite direction constituted 8.74%. In comparison, fewer lncRNAs (1.54%) were located within one kb downstream of protein coding genes. LncRNAs located in the introns of genes were very few (2.17%), as well as lncRNAs with intron containing mRNAs (1.32%). 

LncRNA like mRNA due to alternative splicing events can occur in multiple forms or transcripts. Majority of lncRNAs were composed of one transcript (85.17%) followed by two transcripts (5.45%) (Figure 2C, Supplementary file 3b). Similarly, majority of mRNA transcripts were mostly composed of one transcript (23.47%) followed by 2 (19.23%), 3 (14.48%) and 4 (10.72%) transcripts (Figure 2C, Supplementary file 3b). Some lncRNAs and mRNAs were however composed of >26 transcripts.

### 2.3. Function Enrichment via Potential cis Target Genes of lncRNAs 

**Figure 3:**
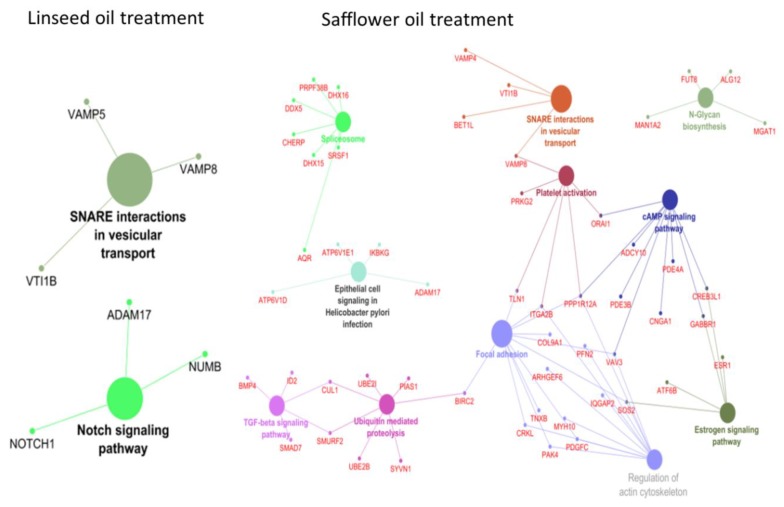
Enriched KEGG pathways for predicted *cis* target genes of lncRNAs.

Correlation analysis of lncRNA and mRNA expression identified 59 and 494 potential *cis* target genes (mRNAs) for lncRNAs in LSO and SFO treatments, respectively (Supplementary file 4). Among them, 38 genes were common to both treatments. A total of 67 (49 biological process gene ontology (GO) terms, 9 cellular components GO terms and 9 molecular functions GO terms) and 15 (12 biological process GO terms, 2 cellular components GO terms and 1 molecular functions GO term) were enriched for *cis* target genes of lncRNAs in SFO and LSO treatments, respectively (Table 1 and Table 2 and Supplementary file 5). The most enriched GO terms were GO:1904375 (regulation of protein localization to cell periphery, *p* = 3.6 × 10^−4^) for LSO and GO:0048294(negative regulation of isotype switching to IgE isotypes, *p* = 2.6 × 10^−3^) for SFO. Moreover, 2 and 11 KEGG pathways were also enriched for LSO and SFO *cis* target genes at uncorrected *p*-value < 0.05, respectively and SNARE interactions in vesicular transport pathway was common to both treatments (Figure 3). The SNARE interaction in vesicular transport pathway was also the most significantly enriched pathway for both LSO and SFO *cis* target genes (Figure 3). 

### 2.4. Effects of Diets Rich in Unsaturated Fatty Acids on lncRNA Expression

Differential gene expression results of the effect of diets on lncRNA expression are shown in Table 3 and Table 4. A total of 32 (11 up-regulated and 21 down-regulated) and 21 (4 up-regulated and 17 down-regulated) lncRNAs were differentially expressed (DE) in LSO and SFO treatments, respectively. Out of this number, seven are known lncRNAs. The highest number of DE lncRNAs was recorded after the first week of supplementation (D+7 vs. D+28) by LSO (21 lncRNAs) and SFO (19 lncRNAs). LncRNAs responded only to LSO (6 DE lncRNAs) at the onset of supplementation (D-14 vs. D+7) while no lncRNA was DE by SFO during this period. Also, few lncRNAs were DE between D-14 and D+28 (10 lncRNAs for LSO and 4 for SFO).

**Figure 4:**
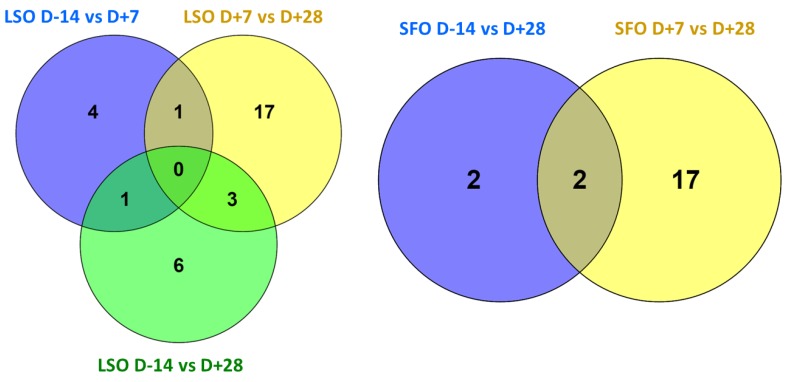
Unique and common significantly (*p*-value BH < 0.1) differentially expressed lncRNAs between periods of comparison.

Comparisons between days for LSO showed that DE lncRNAs were mostly specific to each pair of comparison with only three common DE lncRNAs between D+7 versus D+28 and D-14 versus D+28 (XLOC_049790 (NONBTAT031343.1), XLOC_049791 and XLOC_044269) and one each between D-14 versus D+7 and D+7 versus D+28 (XLOC_004564 (NONBTAT002269.2)) and D-14 versus D+7 and D-14 versus D+28 (XLOC_032807) (Figure 4). For SFO, two lncRNAs (XLOC_053295 (NONBTAT026075.2) and XLOC_049508) were common between D-14 versus D+28 and d D+7 versus D+28 (Figure 4). Two and four *cis* target genes were predicted for DE lncRNAs in LSO and SFO treatments, respectively (Table 5). High correlations were observed between XLOC_007663 and *STARD13* (*r* = 0.89) gene in LSO treatment and between XLOC_020830 and *MMD* gene (*r* = 0.94) in SFO treatment.

### 2.5. Reversed Transcribed PCR (RT-PCR) Verification of the Detection of lncRNA and Real Time Quantitative PCR (qPCR) Verification of the Expression Level of lncRNA

**Figure 5:**
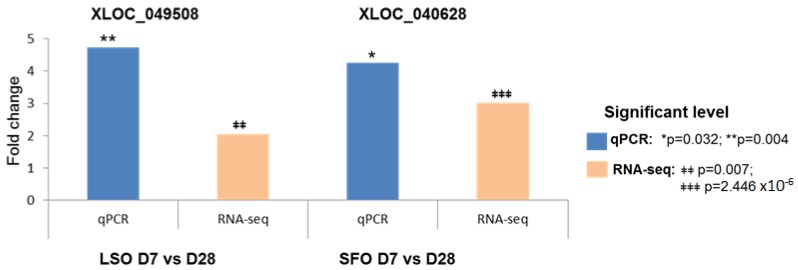
Results of qPCR validation of the expression levels of two lncRNAs and compared to RNA-seq results. LSO = linseed oil; SFO = safflower oil; D = day.

Using RT-PCR, we verified the presence of four lncRNAs (XLOC_003855, XLOC_053295 (NONBTAT026075.2), XLOC_014422 and XLOC_049508) in three different samples (Supplementary file 6). RT-PCR products were of expected sizes (Supplementary file 6), thus confirming RNA-Seq results of lncRNA detection. Moreover, we verified the expression levels of two lncRNAs (XLOC_049508 and XLOC_040628) by real time qPCR (Figure 5). XLOC_049508 and XLOC_040628 were both expressed at >4 fold change, compared to >2 fold change by RNA-seq, thus confirming RNA-seq results.

## 3. Discussion

Previously, we showed a reduction in milk fat yield of 30.38% and 32.42% in response to 5% LSO and 5% SFO, respectively, accompanied by increased concentrations of some monounsaturated and polyunsaturated fatty acids in milk, differential regulation of genes with roles in lipid synthesis/metabolism [53], differential miRNA expression [32] and co-expression network of miRNAs [52]. In the present study, we have characterized the lncRNA repertoire of the bovine mammary gland in response to LSO and SFO. 

A total of 325 known and 4630 novel lncRNAs were identified in this study. Identified lncRNAs were generally less expressed and of smaller sizes compared to mRNA transcripts. Studies on the annotation of human lncRNAs have reported lower expression, smaller size and fewer exons for lncRNAs as compared to mRNAs [54,55] thus supporting our observations. The transcript number per lncRNA gene as compared to mRNA in this study followed the same pattern reported earlier for human [55]. Majority of identified lncRNA transcripts in this study are located in the intergenic regions of protein coding genes (Table S3e). This observation is consistent with previous studies that have reported that lncRNAs are principally located in the intergenic region of genes while a lesser percentage overlap protein coding genes [22,54,55]. Qu and Adelson [22] noted that 67.4% of intergenic bovine ncRNAs had a neighbor gene within 20 kb, with significant number within 5 kb flanking regions of genes. Studies have suggested/demonstrated that lncRNAs may act in *cis* or *trans* to regulate the activities of neighboring genes [56,57,58,59,60,61]. It has been shown that functional clustering of neighbor genes within 5 kb of intergenic ncRNAs resulted in over-representation of regulatory genes [22]. The expression of intergenic lncRNAs was reported to be highly correlated with the expression of neighboring genes within 10 kb [54]. It should be noted that co-expression of lncRNA and mRNA could be due to a true *cis* effect of the lncRNA on the mRNA or due to nearby transcriptional activity of surrounding open chromatin [54,62]. 

Some of the highly expressed lncRNAs identified in this study (13 novel and 15 known) have been detected in bovine tissues, skin and EST data from many developmental stages [20,21,22]. Given that lncRNAs are generally less expressed, the relative high expression levels of the 28 lncRNAs suggest potential roles in the bovine mammary gland. However, validation of their functional significance in the bovine mammary gland merits further investigations. Since it is known that lncRNAs may regulate in *cis* or *trans* the expression of protein coding genes [56,57,58,59,60,61] and since the functions of most bovine lncRNAs are still unknown, we predicted the potential functions of detected lncRNAs via correlated *cis* located mRNAs in the transcriptome data from the same animals. Various GO terms for the potential *cis* target genes of lncRNAs were enriched in different processes (Table 1 and Table 2) which might reflect diverse functions of lncRNAs in the bovine mammary gland. The most enriched GO term for LSO (GO:1904375-regulation of protein localization to cell periphery) does not appear to have a direct functional link with mammary lipid synthesis but it might be important for tissue functioning by modulating the frequency, rate or extent of protein localization to cell periphery. In the SFO treatment, the most enriched term (GO:0048294—negative regulation of isotype switching to IgE isotypes) as well as other enriched GO terms (GO:0002829, GO:0045623 and GO:0045829) showed involvement in immune regulation. The functions of lncRNAs in immunity are well documented [63,64]. Recently, enrichment results by Tong et al. [24] suggest that lncRNAs might play roles in the regulation of immune genes and potentially contribute to disease resistance, such as mastitis in cows. Yang et al. [65] reported involvement of lncRNA H19 in TGF-β1-induced epithelial to mesenchymal transition in bovine epithelial cells and suggested its potential role in immunity and bovine mastitis. In another experiment, Ma et al. [66] reported many lncRNAs that were DE during bovine viral diarrhea virus infection with potential roles in immune functions. As expected, lncRNA target genes were significantly enriched for biological process GO terms involved in regulation of RNA processing (GO:0006396 and GO:1902679) as well as DNA recombination (GO:0045910, GO:0000018). In fact, to perform their functions, lncRNAs might bind to their target genes [67], therefore it is not surprising that the nucleic acids regulation GO terms were enriched. A notable KEGG pathway enriched for LSO treatment was Notch signaling pathway. Notch signaling pathway is important in mammary gland development [68,69]. Previously, we reported that Notch signaling pathway was enriched for target genes of miRNAs in the regulation of milk yield and component traits [70]. It is not clear which specific lncRNAs could be regulating this pathway or how they are involved in the regulation of mammary gland functions. However, the lncRNA HOTAIR has been reported to target the Notch signaling pathway in cervical cancer cells [71]. The SNARE interaction in vesicular transport pathway was significantly enriched for *cis* target genes of lncRNAs in both LSO and SFO treatments. This pathway is important for mediating intracellular destination of transport vesicles [72] as well as membrane fusion [73,74] but it is not clear what role it plays in the regulation of mammary gland functions. Other notable pathways enriched for SFO lncRNA *cis* target genes were cAMP and TGF-β signaling pathways. cAMP was recently identified as an enriched pathway for lncRNA target genes in the bovine mammary gland [24] while TGF-β signaling pathway, known to have important immune functions, was reported as an important pathway for lactation persistency [75] as well as an enriched pathway for target genes of DE miRNAs during a lactation curve [76]. 

Differential gene expression results showed that nutrients rich in unsaturated fatty acids had an effect on lncRNA expression. A comparison of DE lncRNAs between LSO and SFO treatments indicated that more lncRNAs were DE by LSO as compared to SFO and in particular, no lncRNA was DE after one week of SFO supplementation (D-14 vs. D+7). This is similar to our previous observation on mRNA transcriptome of the same data that showed a greater impact of LSO over SFO on gene expression [53]. Also, the mRNA transcriptome data indicated involvement of LSO and SFO DE genes in similar (molecular transport, small molecule biochemistry, lipid metabolism) and different (LSO: cell death and survival, protein synthesis, cellular growth and proliferation and amino acid metabolism; SFO: energy production, cellular movement, cell cycle and carbohydrate metabolism) functions and pathways, which could be due to the different degree of unsaturation of the main fatty acids in LSO and SFO [53]. LSO is rich in α-linolenic acid (3 double bonds in their structure) while SFO is rich in linoleic acid (2 double bonds), which resulted in different intermediates of biohydrogenation in the rumen thus affecting differently the pathways of lipid metabolism and other functions. It is known that the profile of ruminal biohydrogenation intermediates are influenced by the type of diet [77,78] and that pathways related to lipid metabolism have been significantly changed due to diet supplementation [79]. Thus, the observed differential expression of lncRNAs might reflect the change in their functions in response to the type of diet supplement (LSO or SFO). To the best of our knowledge, there are no studies related to lncRNAs expression/function in response to lipid supplements in the mammary gland, so further studies are needed in this area. Moreover, some DE lncRNAs in this study have been previously characterized in bovine [20,22]. These results and our observation suggest regulatory roles of lncRNA in many biological processes including mammary gland functions. Moreover, we also identified six potential *cis* target genes (*KCNF1*, *STARD13*, *BCL6*, *NXPE2*, *HHIPL2* and *MMD*) for DE lncRNAs (Table 5). These genes are involved in lipid metabolism (*STARD13*), molecular transport (*KCNF1*), immune processes/disease (*MMD* and *BCL6*) and in epigenetic processes (*STARD13*). *STARD13* encodes for a member of StAR-related lipid transfer (START) proteins which play important roles in the regulation of intracellular lipid metabolism [80]. MiRNA-125b was shown to induce metastasis in MCF-7 and MDA-MB-231 breast cancer cells through targeting of *STARD13* [81]. The monocyte to macrophage differentiation associated (*MMD*) gene showed the highest level of correlation (*p*-value = 6.54 × 10^−9^) with a lncRNA (XLOC_020830) in SFO treatment. Roles for *MMD* in the positive regulation of ERK and Akt activation and *TNF*-α and nitric oxide production in macrophages have been demonstrated [82]. 

It should be noted that, transcripts of the main proteins (CSN1S1, CSN1S2, CSN2, CSN3, LGB and LALBA and GLYCAM1) in milk constituted 79.45% of the read counts in mammary tissue transcriptome [53] which could impede detection of lowly expressed transcripts. Therefore, a higher sequence read count per sample or depletion of the transcripts of these main proteins might be required to better characterize a class of lowly expressed genes like lncRNAs in mammary tissue. As with many differential gene expression studies, the number of DE genes detected relies on the choice of methodologies (data filtering, read count normalization and comparison between different groups) and selection of methods for correction of multiple testing and threshold for declaration of significant *p*-values. In this study, we chose the Benjamini and Hochberg [83] moderate conservative method for multiple testing which is widely used in the field to avoid losing important DE genes as observed with more conservative methods like Bonferroni correction. It is well documented that the choice of database for enrichment analyses and the methods to test enriched terms also influence results obtained [84,85]. In this study, a hypergeometric test was applied for testing of GO term enrichment using ClueGO [86] platform. This approach has been widely used in the literature and also in our previous study [70]. The potential functions of identified lncRNAs were predicted through inference of the correlation of lncRNA and mRNA expression. However, it is important to note that an observed correlation does not necessarily mean causal relationship. The *cis* target genes predicted based on expression correlation needs to be experimentally functionally verified to confirm their functions. 

## 4. Materials and Methods 

### 4.1. Experimental Animals and Tissue Sampling

Animal care, management and use procedures were according to the national codes of practice for the care and handling of farm animals (http://www.nfacc.ca/codes-of-practice) and approved by the Animal Care and Ethics Committee of Agriculture and Agri-Food Canada (CPA #402, 04 April, 2012). 

The experiment was conducted at the dairy barn of the Sherbrooke Research and Development Centre of Agriculture and Agri-Food Canada. Procedures for animal management and sampling have been reported in our companion papers on the same animals [32,52,53]. In summary, twelve high producing (35 ± 10 kg milk/day) Canadian Holstein cows in mid-lactation (150 ± 50 days in milk) were separated based on parity and days in milk and randomly allocated to one of two treatments: (1) linseed oil treatment (LSO) six cows fed a control diet composed of a total mixed ration of corn and grass silages (50:50) and concentrates supplemented with 5% LSO (on dry matter (DM) bases) and (2) safflower oil treatment (SFO) six cows fed the control diet supplemented with 5% SFO (DM) for 28 days. The treatment period (D+1 to D+28) was preceded by a control period (D-28 to D-1) of 28 days during which time all the animals were on the control diet. The composition of experimental diets is listed in Supplementary file 7. Mammary gland biopsies were harvested from all the animals at three different times during the experimental periods: D-14 (control period), D+7 (7th day after onset of treatment, early treatment period) and D+28 (28th day of treatment, late treatment period) according to an established protocol [87]. Milk samples were collected weekly for the measurement of fat content and fatty acid profiles and the results have been reported [53]. 

### 4.2. RNA Isolation and Sequencing

Total RNA was isolated from 50 mg/biopsy sample with miRNeasy Kit (Qiagen Inc., Toronto, ON, Canada) according to manufacturer’s protocol. Purified RNA was DNase digested with Turbo DNase Kit (Ambion Inc., Foster City, CA, USA) to eliminate DNA contamination. RNA concentration was measured with Nanodrop ND-1000 spectrophotometer (NanoDrop Technologies, Wilmington, DE, USA). The RNA 6000 Nano Labchip Kit (Agilent Technologies, Santa Clara, CA, USA) was used to assess the quality of RNA on an Agilent 2100 Bioanalyzer (Agilent Technologies, Santa Clara, CA, USA). The RNA integrity number of all samples was high and ranged from 7.99 to 9.5.

Thirty-six Libraries (LSO = 18 libraries, SFO = 18 libraries) were each generated from 250 ng total RNA using the TruSeq stranded mRNA Kit (Illumina Inc., San Diego, CA, USA) according to manufacturer’s protocol. The Quant-iT™ PicoGreen^®^ dsDNA Assay Kit (Life Technologies, Burlington, ON, Canada) and the Kapa Illumina GA with the Revised Primers-SYBR Fast Universal Kit (D-Mark Biosciences, Toronto, ON, Canada) were used to quantify generated libraries. Fragment size of libraries was determined on Agilent 2100 Bioanalyzer (Agilent Technologies). The cBot instrument (Illumina Inc.) was used to perform cluster formation on the flow cell. Libraries were multiplexed in equal ratios (six/lane) and sequenced in the form of 50-cycle 100 bp paired-end reads, on a HiSeq 2000 system (Illumina Inc.) running HCS software v2.2.58. After sequencing, demultiplexed FASTQ files were generated by allowing up to one mismatch in the index. Libraries were generated and sequenced by McGill University and Genome Quebec Innovation Centre (MUGQIC, http://gqinnovationcenter.com/).

### 4.3. RNA-Sequence Read Alignment and Identification of lncRNA

RNA-Seq reads from each sample (total of 36) were trimmed using trimmomatic software v0.32 to keep reads longer than 32 bp with a minimum phred score of 30 and to remove adaptor sequences. Reads were then aligned to the bovine genome (UMD3.1) [88] with Tophat (v2.0.11) [89] using default parameters. Uniquely mapped and properly paired reads were assembled with Cufflinks (v2.1.1) [90] and using Ensembl bovine gene annotation release 79. Assembled transcripts from all samples were merged into one using Cuffmerge (Cufflinks v2.1.1) to generate a unique set of all transcripts. Transcripts with a length <200 nt were removed and remaining transcripts compared with Ensembl bovine gene annotation (release 79) to remove transcripts overlapping with known protein coding and noncoding genes (mRNA, tRNA, rRNA, snRNA, snoRNA, miRNA) using Cuffcompare. mRNA transcripts were retained as a separate data set for use in comparing lncRNA expression pattern. Transcripts with class code “i” (an exon falling into an intron of reference transcript), “o” (generic exonic overlap with reference transcripts), “u” (intergenic transcript) and “x” (exonic overlap with reference transcript on the opposite strand) were retained. Retained transcripts were evaluated for their coding potentials using Coding-Non-Coding Index (CNCI) program [91]. CNCI is effective for distinguishing protein-coding and non-coding nucleotide sequences by profiling adjoining nucleotide triplets. Those transcripts assigned with a negative CNCI score were classified as candidate non-coding transcripts. The coding potential of candidate non-coding transcripts was further assessed with Coding Potential Assessment Tool (CPAT) [92]. CPAT was trained with available bovine known protein-coding transcripts from Ensembl bioMart and bovine non-coding sequences (NONCODE2016) [93] to build a logistic regression model. The resulting CPAT coding probability score for the transcripts ranges between 0 and 1 with a higher score indicating a higher coding potential. We chose a cut-off value of 0.4 for determining protein coding probability. 

The remaining transcripts were then blasted against the Swiss-prot database to remove those with a hit (*e* value < 1 × 10^−5^) using usearch [94]. Retained transcripts were compared with known bovine lncRNA annotation from NONCODE2016 database [93,95]. Those transcripts with class codes of “=” (complete match with reference transcript), “c” (contained in reference transcript) and “j” (novel isoform of reference transcript) were classified as known bovine lncRNA whereas the rest were classified as novel lncRNA. The identified lncRNA were further classified into 11 classes with the reference of Ensembl bovine protein coding gene annotation. 

### 4.4. Gene Ontology and Pathways Enrichment for lncRNA cis Target Genes 

Since lncRNAs can *cis* regulate mRNAs [56,57,58,59,60,61], we performed enrichments for lncRNA *cis* regulatory functions by using mRNA transcriptome data obtained from the same animals [53]. For each lncRNA, Pearson correlation of its expression value with that of each mRNA was calculated. The closest coding genes within 50 kb upstream and downstream of lncRNAs were mined using BEDTools v2.25.0 program [96]. The genes were considered potential *cis* target genes of lncRNAs if in addition to their location (within a 50 kb window upstream or downstream of lncRNAs) they had a Pearson correlation of >0.7 with lncRNAs. 

These *cis* target genes were submitted to the ClueGo plugin [86] in Cytoscape [97] for GO term and KEGG pathways enrichment analysis. Enriched pathways and GO terms were tested using a hypergeometric test which estimates enrichment by evaluating the overlap between genes in a given gene set (input gene list) followed by annotating genes to a GO term or pathway. The null hypothesis was ‘the annotated GO term or pathway was irrelevant to the input list’. The *p*-value measures the significance of enrichment derived from the tail probability of observing numbers of DE genes annotated to the GO term or pathway. Enriched GO terms were declared significant at Benjamini and Hochberg [83] adjusted *p*-value ≤ 0.05 while a lower threshold at uncorrected *p*-value < 0.05 were considered significant for KEGG pathways enrichment. 

### 4.5. LncRNA Expression and Differential Gene Expression Analysis

The expression of identified lncRNAs (known and novel) was quantified in each sample using HTSeq-count (version 0.6.1p1) with default settings (-s reverse). The raw read counts of retained transcripts of all libraries were then imported into DESeq2 [98] to identify differentially expressed lncRNAs. DESeq2 calculates a size factor for each sample to correct for library size and RNA composition bias. Those lncRNAs with DESeq2 normalized counts ≥5 in at least 10% of our libraries were considered as truly expressed. Significantly differentially expressed lncRNAs were defined as having a Benjamini and Hochberg adjusted *p*-value < 0.1. The expression level of each lncRNA was determined as FPKM. To further illustrate the functions of lncRNA in the nutrient effects on mammary gland, the same procedure for enrichments using Clue GO was applied for *cis* target genes of lncRNAs DE by treatments. 

### 4.6. Reversed Transcribed (RT)-PCR

Reversed transcribed- PCR was performed to verify the presence of lncRNAs detected by RNA sequencing. Primers for four randomly selected lncRNAs (XLOC_003855, XLOC_053295 (NONBTAT026075.2), XLOC_014422 and XLOC_049508) were designed using Integrated DNA Technologies Assay tool (https://www.idtdna.com/scitools/Applications/RealTimePCR/). The gene-specific primers used for detecting lncRNAs are shown in Supplementary file 6. Reverse transcription was performed with SuperScript™ II Reverse Transcriptase (Life Technologies Inc., Carlsbad, CA, USA), using 500 ng of the same total RNA used in RNA sequencing. cDNA templates were amplified in three different samples by PCR using Crimson Taq DNA polymerase (New England BioLabs, Whitby, ON, Canada). All PCR reactions were performed using the Veriti 96 well thermal cycler (Applied Biosystems, Foster City, CA, USA). An initial PCR gradient was done to determine the best annealing temperature for each primer pair. Thermal cycling condition was composed of an initial denaturation at 95 °C for 4 min followed by 45 cycles of 30 s denaturation at 95 °C, 1 min annealing at 52 °C and elongation at 72 °C for 30 s. The final extension step was done at 72 °C for 5 min. The PCR products (~300–600 bp) were run on 1.5% agarose gel and visualized with Fusion FX (Birch House, Brambleside, Uckfield, UK). A 100bp ladder was run alongside the samples. 

### 4.7. Real-Time qPCR Verification of lncRNA Expression

Validation of the RNA-seq expression levels of two randomly selected DE lncRNAs was done using real-time quantitative PCR. Reverse transcription was performed with the SuperScript™ III Reverse Transcriptase (Life Technologies), using aliquots (1 μg) of the same total RNA used in RNA-seq. The cDNA samples were diluted to 20 ng/μL. Transcript-specific primers were designed using Integrated DNA Technologies RealTime qPCR Assay tool (https://www.idtdna.com/scitools/Applications/RealTimePCR/) (Supplementary file 8). Real-time PCR reaction mix was composed of 5 µL Power SYBR^®^ Green PCR Master Mix (Life Technologies Inc., Burlington, ON, Canada), 3 µL cDNA, 300 nM of each forward and reverse primers and 0.1 U AmpErase^®^ Uracil N-Glycosylase (UNG, Life Technologies, Carlsbad, CA, USA). QPCR reactions were performed using the StepOnePlus™ Real-Time PCR System (Life Technologies). The thermal cycling conditions were composed of a step for UNG treatment at 25 °C for 5 min followed by an initial denaturation/activation step at 95 °C for 10 min, 45 cycles at 95 °C for 30 s, 60 °C for 60 s. The experiments were carried out in triplicate for each data point. The relative quantification of gene expression was determined using the 2^−ΔΔ*C*t^ method [99]. The fold change in gene expression was obtained following normalization to two reference genes, *RPS15* and *GAPDH*. The stability of these reference genes have been previously tested [53].

## 5. Conclusions

A total of 4955 lncRNAs (325 known and 4630 novel) were identified including 32 (11 up-regulated and 21 down-regulated) and 21 (4 up-regulated and 17 down-regulated) lncRNAs differentially expressed in LSO and SFO treatments, respectively. The impact of LSO on lncRNA expression was early and also more potent as compared to SFO. GO and pathway analyses of lncRNA *cis* target genes suggest regulatory roles for lncRNAs in mammary gland functions, immune functions and metabolism/regulation of nucleic acid processes in the mammary gland. Furthermore, lncRNAs DE by LSO or SFO suggest potential regulatory roles in mammary lipid metabolism and synthesis of lipid/fatty acid. The identified lncRNAs will further enrich the catalogue of bovine lncRNAs and will contribute in the understanding of mammary gland functions and biology.

## Figures and Tables

**Table 1:** Gene ontologies enriched for *cis* target genes of lncRNAs in LSO treatments.

GOID	GO Term	Ontology Source	*p*_Value	*p*_FDR
GO:1904375	Regulation of protein localization to cell periphery	GO_BP	8.40 × 10^−5^	3.60 × 10^−4^
GO:1903729	Regulation of plasma membrane organization	GO_BP	1.10 × 10^−4^	3.80 × 10^−4^
GO:1903076	Regulation of protein localization to plasma membrane	GO_BP	7.20 × 10^−5^	4.60 × 10^−4^
GO:0060412	Ventricular septum morphogenesis	GO_BP	2.00 × 10^−4^	5.40 × 10^−4^
GO:0048546	Digestive tract morphogenesis	GO_BP	4.40 × 10^−4^	8.30 × 10^−4^
GO:0030858	Positive regulation of epithelial cell differentiation	GO_BP	6.90 × 10^−4^	1.00 × 10^−3^
GO:0060411	Cardiac septum morphogenesis	GO_BP	1.00 × 10^−3^	1.30 × 10^−3^
GO:0003281	Ventricular septum development	GO_BP	1.00 × 10^−3^	1.30 × 10^−3^
GO:0055006	Cardiac cell development	GO_BP	1.20 × 10^−3^	1.40 × 10^−3^
GO:0048663	Neuron fate commitment	GO_BP	1.30 × 10^−3^	1.40 × 10^−3^
GO:0048708	Astrocyte differentiation	GO_BP	1.40 × 10^−3^	1.40 × 10^−3^
GO:0002040	Sprouting angiogenesis	GO_BP	1.40 × 10^−3^	1.40 × 10^−3^
GO:0055038	Recycling endosome membrane	GO_CC	4.30 × 10^−5^	5.50 × 10^−4^
GO:0031201	SNARE complex	GO_CC	6.60 × 10^−4^	1.00 × 10^−3^
GO:0005484	SNAP receptor activity	GO_MF	2.20 × 10^−4^	4.90 × 10^−4^

GO_BP: Biological process GO term, GO_CC: Cellular component GO term, GO_MF: Molecular function GO term.

**Table 2:** Gene ontologies enriched for *cis* target genes of lncRNAs in SFO treatments.

GOID	GO Term	Ontology Source	*p*_Value	*p*_FDR
GO:0048294	Negative regulation of isotype switching to IgE isotypes	GO_BP	1.50 × 10^−5^	2.60 × 10^−3^
GO:0045910	Negative regulation of DNA recombination	GO_BP	1.10 × 10^−5^	4.20 × 10^−3^
GO:0045829	Negative regulation of isotype switching	GO_BP	5.90 × 10^−5^	7.00 × 10^−3^
GO:0010633	Negative regulation of epithelial cell migration	GO_BP	8.60 × 10^−5^	7.70 × 10^−3^
GO:0006396	RNA processing	GO_BP	1.00 × 10^−4^	7.70 × 10^−3^
GO:0002262	Myeloid cell homeostasis	GO_BP	2.20 × 10^−4^	1.10 × 10^−2^
GO:0000018	Regulation of DNA recombination	GO_BP	3.20 × 10^−4^	1.10 × 10^−2^
GO:0030218	Erythrocyte differentiation	GO_BP	3.00 × 10^−4^	1.10 × 10^−2^
GO:1902679	Negative regulation of RNA biosynthetic process	GO_BP	2.10 × 10^−4^	1.20 × 10^−2^
GO:0048289	Isotype switching to IgE isotypes	GO_BP	4.90 × 10^−4^	1.50 × 10^−2^
GO:0048293	Regulation of isotype switching to IgE isotypes	GO_BP	4.90 × 10^−4^	1.50 × 10^−2^
GO:0045646	Regulation of erythrocyte differentiation	GO_BP	6.20 × 10^−4^	1.70 × 10^−2^
GO:0034101	Erythrocyte homeostasis	GO_BP	6.20 × 10^−4^	1.80 × 10^−2^
GO:0002638	Negative regulation of immunoglobulin production	GO_BP	7.70 × 10^−4^	1.80 × 10^−2^
GO:0045654	Positive regulation of megakaryocyte differentiation	GO_BP	7.70 × 10^−4^	1.80 × 10^−2^
GO:0045892	Negative regulation of transcription, DNA-templated	GO_BP	7.60 × 10^−4^	1.90 × 10^−2^
GO:0060840	Artery development	GO_BP	9.60 × 10^−4^	2.00 × 10^−2^
GO:0016071	mRNA metabolic process	GO_BP	1.20 × 10^−3^	2.40 × 10^−2^
GO:1903706	Regulation of hemopoiesis	GO_BP	1.60 × 10^−3^	2.70 × 10^−2^
GO:0010720	Positive regulation of cell development	GO_BP	1.80 × 10^−3^	2.80 × 10^−2^
GO:0060602	Branch elongation of an epithelium	GO_BP	1.90 × 10^−3^	2.80 × 10^−2^
GO:0002829	Negative regulation of type 2 immune response	GO_BP	2.10 × 10^−3^	3.00 × 10^−2^
GO:0003158	Endothelium development	GO_BP	2.30 × 10^−3^	3.20 × 10^−2^
GO:0010632	Regulation of epithelial cell migration	GO_BP	2.50 × 10^−3^	3.20 × 10^−2^
GO:0006260	DNA replication	GO_BP	3.70 × 10^−3^	3.40 × 10^−2^
GO:0002064	Epithelial cell development	GO_BP	2.70 × 10^−3^	3.40 × 10^−2^
GO:0060442	Branching involved in prostate gland morphogenesis	GO_BP	2.80 × 10^−3^	3.40 × 10^−2^
GO:0045623	Negative regulation of T-helper cell differentiation	GO_BP	2.80 × 10^−3^	3.40 × 10^−2^
GO:0045648	Positive regulation of erythrocyte differentiation	GO_BP	3.60 × 10^−3^	3.40 × 10^−2^
GO:0035561	Regulation of chromatin binding	GO_BP	3.10 × 10^−3^	3.60 × 10^−2^
GO:1903708	Positive regulation of hemopoiesis	GO_BP	3.40 × 10^−3^	3.60 × 10^−2^
GO:0045620	Negative regulation of lymphocyte differentiation	GO_BP	3.20 × 10^−3^	3.70 × 10^−2^
GO:0070076	Histone lysine demethylation	GO_BP	4.70 × 10^−3^	3.80 × 10^−2^
GO:1903573	Negative regulation of response to endoplasmic reticulum stress	GO_BP	4.90 × 10^−3^	3.80 × 10^−2^
GO:1902105	Regulation of leukocyte differentiation	GO_BP	4.40 × 10^−3^	3.80 × 10^−2^
GO:0030968	Endoplasmic reticulum unfolded protein response	GO_BP	5.60 × 10^−3^	4.20 × 10^−2^
GO:0016577	Histone demethylation	GO_BP	6.10 × 10^−3^	4.30 × 10^−2^
GO:1902106	Negative regulation of leukocyte differentiation	GO_BP	6.20 × 10^−3^	4.30 × 10^−2^
GO:0016447	Somatic recombination of immunoglobulin gene segments	GO_BP	5.90 × 10^−3^	4.30 × 10^−2^
GO:0006349	Regulation of gene expression by genetic imprinting	GO_BP	6.60 × 10^−3^	4.50 × 10^−2^
GO:0002467	Germinal center formation	GO_BP	6.60 × 10^−3^	4.50 × 10^−2^
GO:0045064	T-helper 2 cell differentiation	GO_BP	6.60 × 10^−3^	4.50 × 10^−2^
GO:0045652	Regulation of megakaryocyte differentiation	GO_BP	6.60 × 10^−3^	4.50 × 10^−2^
GO:0001568	Blood vessel development	GO_BP	7.20 × 10^−3^	4.70 × 10^−2^
GO:0034620	Cellular response to unfolded protein	GO_BP	7.10 × 10^−3^	4.70 × 10^−2^
GO:0006482	Protein demethylation	GO_BP	7.70 × 10^−3^	4.80 × 10^−2^
GO:0050869	Negative regulation of B cell activation	GO_BP	7.70 × 10^−3^	4.80 × 10^−2^
GO:2000241	Regulation of reproductive process	GO_BP	8.20 × 10^−3^	4.90 × 10^−2^
GO:0048872	Homeostasis of number of cells	GO_BP	7.70 × 10^−3^	4.90 × 10^−2^
GO:0031252	Cell leading edge	GO_CC	8.30 × 10^−4^	1.80 × 10^−2^
GO:0031256	Leading edge membrane	GO_CC	1.50 × 10^−3^	2.50 × 10^−2^
GO:0001726	Ruffle	GO_CC	1.40 × 10^−3^	2.60 × 10^−2^
GO:0042581	Specific granule	GO_CC	3.80 × 10^−3^	3.30 × 10^−2^
GO:0032039	Integrator complex	GO_CC	3.50 × 10^−3^	3.50 × 10^−2^
GO:0031253	Cell projection membrane	GO_CC	3.40 × 10^−3^	3.70 × 10^−2^
GO:0098858	Actin-based cell projection	GO_CC	4.40 × 10^−3^	3.80 × 10^−2^
GO:0005902	Microvillus	GO_CC	5.80 × 10^−3^	4.30 × 10^−2^
GO:0055037	Recycling endosome	GO_CC	6.60 × 10^−3^	4.40 × 10^−2^
GO:0051731	Polynucleotide 5′-hydroxyl-kinase activity	GO_MF	2.80 × 10^−4^	1.20 × 10^−2^
GO:0008134	Transcription factor binding	GO_MF	1.40 × 10^−3^	2.60 × 10^−2^
GO:0051020	GTPase binding	GO_MF	3.60 × 10^−3^	3.40 × 10^−2^
GO:0019787	Ubiquitin-like protein transferase activity	GO_MF	3.50 × 10^−3^	3.60 × 10^−2^
GO:0030374	Ligand-dependent nuclear receptor transcription coactivator activity	GO_MF	3.30 × 10^−3^	3.70 × 10^−2^
GO:0005089	Rho guanyl-nucleotide exchange factor activity	GO_MF	4.80 × 10^−3^	3.80 × 10^−2^
GO:0060589	Nucleoside-triphosphatase regulator activity	GO_MF	4.70 × 10^−3^	3.90 × 10^−2^
GO:0035591	Signaling adaptor activity	GO_MF	5.80 × 10^−3^	4.30 × 10^−2^
GO:0031267	Small GTPase binding	GO_MF	7.80 × 10^−3^	4.80 × 10^−2^

GO_BP: Biological process GO term, GO_CC: Cellular component GO term, GO_MF: Molecular function GO term.

**Table 3:** Differentially expressed lncRNAs in response to dietary supplementation with 5% linseed oil.

Periods of Comparison	Known lncRNA Notation	Chr	Chr Start..End	Nearest Gene	FC	log2FC	*p*-Value	Padj
**D-14 vs. D+7**								
XLOC_044813	NONBTAT030934.1	6	17675271..17680447	-	-	−1.459	2.247 × 10^−7^	0.0010
	NONBTAG014563.2				2.749			
XLOC_032807	New	26	33011966..33019072	-	2.310	1.208	7.345 × 10^−7^	0.0017
XLOC_041145	NONBTAT020143.2	4	95417314..95613931	-	-	−1.131	3.393 × 10^−6^	0.0051
	NONBTAG013424.2				2.190			
XLOC_021427	New	19	31640546..31641078	-	−2.378	−1.250	8.409 × 10^−6^	0.0095
XLOC_021420	New	19	31604279..31663126	-	−2.258	−1.175	3.207 × 10^−5^	0.0289
XLOC_004564	NONBTAT002269.2	10	49527965..49605076	RORA (ENSBTAT00000021144)	1.720	0.782	0.0001	0.0769
	NONBTAG013424.2							
**D+7 vs. D+28**								
XLOC_050004	New	8	64796589..64820744	-	-	−1.111	4.666 × 10^−8^	0.0001
					2.160			
XLOC_004564	NONBTAT002269.2	10	49527965..49605076	RORA (ENSBTAT00000021144)	-	−0.886	1.084 × 10^−5^	0.0065
	NONBTAG001608.2				1.848			
XLOC_018587	New	18	11048799..11055932	CRISPLD2 (ENSBTAT00000028221)	-	−0.814	8.899 × 10^−6^	0.0065
					1.758			
XLOC_049508	New	8	22729811..22734496	ENSBTAG00000047195	-	−1.045	8.973 × 10^−6^	0.0065
				(ENSBTAT00000048617)	2.063			
XLOC_026857	New	21	31732594..31860453	FBXO22 (ENSBTAT00000003665)	-	−0.557	2.408 × 10^−5^	0.0116
					1.471			
XLOC_024438	New	2	134829905..134832425	-	-	−0.865	7.330 × 10^−5^	0.0196
					1.821			
XLOC_039327	New	3	114773040..114828419	-	-	−0.762	6.304 × 10^−5^	0.0196
					1.696			
XLOC_049790	NONBTAT031343.1	8	42264400..42279290	-	1.873	0.905	6.640 × 10^−5^	0.0196
	NONBTAG022051.1							
XLOC_049791	New	8	42273319..42275306	-	2.172	1.119	6.569 × 10^−5^	0.0196
XLOC_049767	New	8	42141722..42245895	-	1.911	0.934	8.906 × 10^−5^	0.0214
XLOC_049792	NONBTAT031344.1	8	42279395..42321282	-	2.032	1.023	0.0002	0.0410
	NONBTAG016235.2							
XLOC_011302	New	14	84116708..84118510	-	2.035	1.025	0.0002	0.0466
XLOC_030043	New	23	36290019..36292016	-	1.691	0.758	0.0003	0.0467
XLOC_004276	New	10	26691672..26693523	-	1.657	0.729	0.0003	0.0590
XLOC_007663	New	12	27541269..28034473	STARD13 (ENSBTAT00000029081)	-	−0.411	0.0004	0.0592
					1.330			
XLOC_005960	New	11	86699810..86710266	-	-	−0.868	0.0006	0.0825
					1.825			
XLOC_014482	New	16	52617661..52619004	-	-	−0.708	0.0006	0.0825
					1.634			
XLOC_050157	New	8	77681842..77683706	-	-	−0.956	0.0006	0.0825
					1.940			
XLOC_051249	New	8	84443959..84447116	-	-	−0.751	0.0007	0.0825
					1.683			
XLOC_040832	New	4	66327795..66329807	-	-	−0.922	0.0008	0.0913
					1.895			
XLOC_044269	New	5	100899101..100938587	-	-	−0.504	0.0009	0.0985
					1.418			
**D-14 vs. D+28**								
XLOC_032807	New	26	33011966..33019072	-	2.178	1.123	3.826 × 10^−6^	0.0023
XLOC_040082	New	4	93460873..93469656	HIG2 (ENSBTAT00000045181)	2.310	1.208	2.246 × 10^−6^	0.0023
XLOC_044264	New	5	100888960..100892632	-	-	−1.056	4.000 × 10^−6^	0.0023
					2.079			
XLOC_044269	New	5	100899101..100938587	-	-	−0.613	4.937 × 10^−5^	0.0152
					1.529			
XLOC_045228	New	6	87225278..87228405	-	-	−0.924	4.182 × 10^−5^	0.0152
					1.897			
XLOC_053316	New	Mt	2..360	-	-	−0.978	5.330 × 10^−5^	0.0152
					1.970			
XLOC_049790	NONBTAT031343.1	8	42264400..42279290	-	1.813	0.858	0.0001	0.0349
	NONBTAG022051.1							
XLOC_054333	New	X	123683291..124283250	ENSBTAG00000048092	1.580	0.660	0.0004	0.0847
				(ENSBTAT00000030016)				
XLOC_002555	New	1	153149175..153164789	-	-	−0.766	0.0006	0.0973
					1.701			
XLOC_049791	New	8	42273319..42275306	-	1.957	0.969	0.0005	0.0973

**Table 4:** Differentially expressed lncRNAs in response to dietary supplementation with 5% safflower oil.

Periods of Comparison	LncRNA Type	Chr	Chr Start..End	Nearest Gene	FC	log2FC	*p*-Value	Padj
**D-14 vs. D+28**								
XLOC_053295	NONBTAT026075.2	Mt	1453..3023	ENSBTAG00000043570	1.683	0.751	2.491 × 10^−6^	0.0107
	NONBTAG017440.2			(ENSBTAT00000060540)				
XLOC_014422	New	16	50833181..50845563	ARHGEF16 (ENSBTAT00000027769)	-	−1.014	7.395 × 10^−6^	0.0159
					2.020			
XLOC_033615	New	27	24828725..24847714	-	1.709	0.773	2.105 × 10^−5^	0.0302
XLOC_049508	New	8	22729811..22734496	ENSBTAG00000047195	-	−0.895	4.575 × 10^−5^	0.0492
				(ENSBTAT00000048617)	1.860			
**D+7 vs. D+28**								
XLOC_040628	New	4	36035624..36063375	-	-	−1.601	9.914 × 10^−10^	2.446 × 10^−6^
					3.034			
XLOC_039658	New	4	36060986..36063955	-	-	−1.005	3.385 × 10^−5^	0.0417
					2.007			
XLOC_001923	New	1	80169821..80179076	-	-	−0.603	0.0002	0.0828
					1.519			
XLOC_005093	New	10	100872512..100938144	-	-	−0.505	0.00030	0.0828
					1.419			
XLOC_012186	New	15	25534554..25535977	-	-	−0.973	0.0003	0.0828
					1.963			
XLOC_014185	New	16	26739288..26747603	TAF1A (ENSBTAT00000017928)	-	−0.610	0.0005	0.0828
					1.526			
XLOC_016131	New	17	64537633..64544131	-	-	−0.721	0.0005	0.0828
					1.648			
XLOC_020830	NONBTAT028906.1	19	5793670..5835852	MMD (ENSBTAT00000000244)	1.396	0.481	0.0004	0.0828
	NONBTAG019735.1							
XLOC_034163	New	27	41333596..41335357	-	-	−0.944	0.0003	0.0828
					1.924			
XLOC_040082	New	4	93460873..93469656	HIG2 (ENSBTAT00000045181)	1.806	0.853	0.0005	0.0828
XLOC_042624	New	5	82039036..82042254	-	-	−0.887	0.0002	0.0828
					1.849			
XLOC_044264	New	5	100888960..100892632	-	-	−0.834	0.0004	0.0828
					1.783			
XLOC_049508	New	8	22729811..22734496	ENSBTAG00000047195	-	−0.788	0.0003	0.0828
				(ENSBTAT00000048617)	1.727			
XLOC_052993	New	9	95309003..95312277	-	-	−0.952	0.0003	0.0828
					1.935			
XLOC_053295	NONBTAT026075.2	Mt	1453..3023	ENSBTAG00000043570	1.476	0.562	0.0004	0.0828
	NONBTAG017440.2			(ENSBTAT00000060540)				
XLOC_015543	New	16	67180319..67190790	-	-	−0.429	0.0006	0.0878
					1.346			
XLOC_047068	New	7	27353120..27714937	CTXN3 (ENSBTAT00000044060)	-	−0.748	0.0006	0.0878
					1.679			
XLOC_002568	New	1	153860742..153955586	ENSBTAG00000044519	-	−0.648	0.0007	0.0971
				(ENSBTAT00000061952)	1.567			
XLOC_043291	New	5	6768479..6777191	-	-	−0.778	0.0007	0.0971
					1.715			

**Table 5:** Potential *cis* target genes for differentially expressed lncRNAs in linseed oil and safflower oil treatments.

Treatment	LncRNA	Gene	Correlation	*p*-Value	Chromosome	Gene.Start	Gene.End
Linseed oil	XLOC_005960	KCNF1	0.821379	2.93 × 10^−5^	11	86759338	86761647
Linseed oil	XLOC_007663	STARD13	0.894839	5.38 × 10^−7^	12	27866074	28033238
Safflower oil	XLOC_001923	BCL6	0.797951	7.25 × 10^−5^	1	80179482	80202388
Safflower oil	XLOC_012186	NXPE2	0.822692	2.77 × 10^−5^	15	25515542	25524857
Safflower oil	XLOC_014185	HHIPL2	0.739728	0.00045	16	26713225	26741364
Safflower oil	XLOC_020830	MMD	0.940505	6.54 × 10^−9^	19	5819716	5840124

## Supplementary Materials

Supplementary materials can be found at http://www.mdpi.com/1422-0067/19/11/3610/s1. Supplementary file 1: Identified lncRNA read counts: (a) Novel lncRNAs, (b) gtf file of novel lncRNAs, (c) Known lncRNAs and (d) gtf file of known lncRNAs; Supplementary file 2: Chromosomal distribution of identified novel and known lncRNA genes; Supplementary file 3: (a) Length distribution of identified lncRNAs; (b) transcript distribution of identified lncRNAs compared with transcript distribution of protein coding genes; (c) Class distribution of lncRNAs. Supplementary file 4: *Cis* target genes of identified lncRNAs. Supplementary file 5: Gene ontology and KEGG pathways enriched for *cis* target genes of lncRNAs; Supplementary file 6: (A) LncRNA genes detected by RT-PCR in three different samples (1, 2, 3). PCR products were of expected sizes; 349 bps for XLOC_003855 (i), 574 bps for XLOC_053295 (ii), 441 bps for XLOC_014422 (iii) and 319 bps for XLOC_049508 (iv). M = 100 bp ladder. (B) Primer sequences used in RT-PCR. Supplementary file 7: Ingredients and chemical composition of the experimental diets. Supplementary file 8: Primer sequences used in real time quantitative PCR (qPCR).

## Availability of supporting data

The sequence data has been submitted to Gene Expression Omnibus database (BioProject ID: PRJNA301777) and is available through this link: http://www.ncbi.nlm.nih.gov/bioproject/301777.

## Abbreviations

ANGPTL4	Angiopoietin like 4
BCL6	B-cell CLL/lymphoma 6
bp	Base pair
BTA	Bovine chromosome
CLA	Conjugated linoleic acid
CNCI	Coding-Non-Coding Index
CPAT	Coding Potential Assessment Tool
DE	Differentially expressed
DM	Dry matter
ERK	Extracellular signal–regulated kinase
EST	Expressed sequence tag
FPKM	Fragments per kilo base of transcript per million mapped reads
*GAPDH*	Glyceraldehyde-3-phosphate dehydrogenase
GO	Geno ontology
HHIPL2	HHIP like 2
kb	Kilo base pairs
KCNF1	Potassium voltage-gated channel modifier subfamily F member 1
KEGG	Kyoto encyclopedia of genes and genomes
LncRNA	Long non-coding RNA
LSO	Linseed oil
MALAT1	Metastasis associated lung adenocarcinoma transcript 1
Mb	Mega base pairs
miRNA	Micro RNA
MMD	Macrophage differentiation associated
mRNA	Messenger RNA
Mt	Mitochondria
ncRNA	Non-coding RNA
nt	Nucleotides
NXPE2	Neurexophilin and PC-esterase domain family member 2
PINC	Pregnancy-induced non coding RNA
QTL	Quantitative trait loci
rRNA	ribosomal RNA
*RPS15*	Ribosomal protein S15
RT-PCR	Reverse transcription polymerase chain reaction
SFO	Safflower oil
snoRNA	Small nucleolar RNA
snRNA	Small nuclear RNA
STARD13	StAR related lipid transfer domain containing 13
TNF-α	Tumor necrosis factor-alpha
tRNA	Transfer RNA
XIST	X inactive specific transcript
Zfas1	ZNFX1 antisense RNA 1
ZNFX1	Zinc finger NFX1-type containing 1
