# Supplementary material for: Transcriptome Analysis of Long Non-Coding RNA in the Bovine Mammary Gland Following Dietary Supplementation with Linseed Oil and Safflower Oil
Source: Int J Mol Sci. 2018 Nov 15;19(11):3610. doi: 10.3390/ijms19113610 (PMC6274745; doi:10.3390/ijms19113610)
Supplement: Supplementary file 1 [file ijms-19-03610-s001.zip › Supplementary File 8_lncRNA_qPCR_primer_sequences.docx]

**Additional file 8: Primer sequences used in qPCR validation of RNA-sequencing data**

| Target gene | Accession number | Primer | Concentration (nM) | Sequence | Amplicon length |
| --- | --- | --- | --- | --- | --- |
| XLOC_049508 |  | Forward | 300 | 5’- CCC AGT TGC TAG AAG ACT AAC C -3’ | 319 |
|  |  | Reverse | 300 | 5’- CCA GTA CTC AGA GCT GAT GAA G -3’ |  |
| XLOC_040628 |  | Forward | 300 | 5’- GGC ACA AAA CTG GGT AGC TCT -3’ | 111 |
|  |  | Reverse | 300 | 5’- ATG TTG ACA AAC TGA CTA TGG CAC -3’ |  |
| GAPDH | NM_001034034.2 | Forward | 300 | 5’-TGG AAA GGC CAT CAC CAT CT-3’ | 62 |
|  |  | Reverse | 300 | 5’-CCC ACT TGA TGT TGG CAG-3’ |  |
| RPS15 | NM_001024541.2 | Forward | 300 | 5’-GAT CAT TCT ACC CGA GAT GGT G-3’ | 127 |
|  |  | Reverse | 300 | 5’-GGG CTT GTA AGT GAT GGA GAA-3’ |  |
