# Supplementary material for: Transcriptome Analysis of Long Non-Coding RNA in the Bovine Mammary Gland Following Dietary Supplementation with Linseed Oil and Safflower Oil
Source: Int J Mol Sci. 2018 Nov 15;19(11):3610. doi: 10.3390/ijms19113610 (PMC6274745; doi:10.3390/ijms19113610)
Supplement: Supplementary file 1 [file ijms-19-03610-s001.zip › Supplementary File 7_Ingredients and composition.docx]

**Additional File 7***:* Ingredients and chemical composition of the experimental diets

|  | | | | |
| --- | --- | --- | --- | --- |
| Item (% DM, unless otherwise noted) | Control diet |  | Unsaturated fatty acid enriched treatments | |
|  |  |  | Safflower oil treatment | Linseed oil treatment |
| Ingredients |  |  |  |  |
| Chopped hay | 3.3 |  | 3.3 | 3.3 |
| Corn silage | 27.3 |  | 27.5 | 27.5 |
| Grass silage | 27.5 |  | 27.6 | 27.6 |
| Corn grain, ground | 22.7 |  | 16.9 | 16.9 |
| Safflower oil | - |  | 5.0 | - |
| Linseed oil | - |  | - | 5.0 |
| Soybean meal | 15.7 |  | 16.2 | 16.2 |
| Calcium carbonate | 0.5 |  | 0.6 | 0.6 |
| Protein supplements^1^ | 1.4 |  | 1.4 | 1.4 |
| Mixed minerals | 1.5 |  | 1.5 | 1.5 |
| Chemical composition | |  |  |  |
| Acid detergent fiber, | 19.4 |  | 20.2 | 20.2 |
| Neutral detergent fiber | 31.1 |  | 30.9 | 30.9 |
| Net energy level_,_ Mcal^2^ /kg of DM | 1.55 |  | 1.75 | 1.75 |
| Crude protein | 17.4 |  | 16.7 | 16.7 |
| Starch | 20.14 |  | 20.47 | 19.1 |
| Protein | 42 |  | 35.0 | 35.0 |
| Dry matter | 44.2 |  | 44.2 | 44.2 |

^1^Protein supplements= 30% corn gluten feed, 30% corn distillers grain, 20% canola meal and 20% heat treated soybean meal.

^2^Mcal=Mega calorie.
