# Supplementary figures and images for: Transcriptome Analysis of Long Non-Coding RNA in the Bovine Mammary Gland Following Dietary Supplementation with Linseed Oil and Safflower Oil
Source: Int J Mol Sci. 2018 Nov 15;19(11):3610. doi: 10.3390/ijms19113610 (PMC6274745; doi:10.3390/ijms19113610)

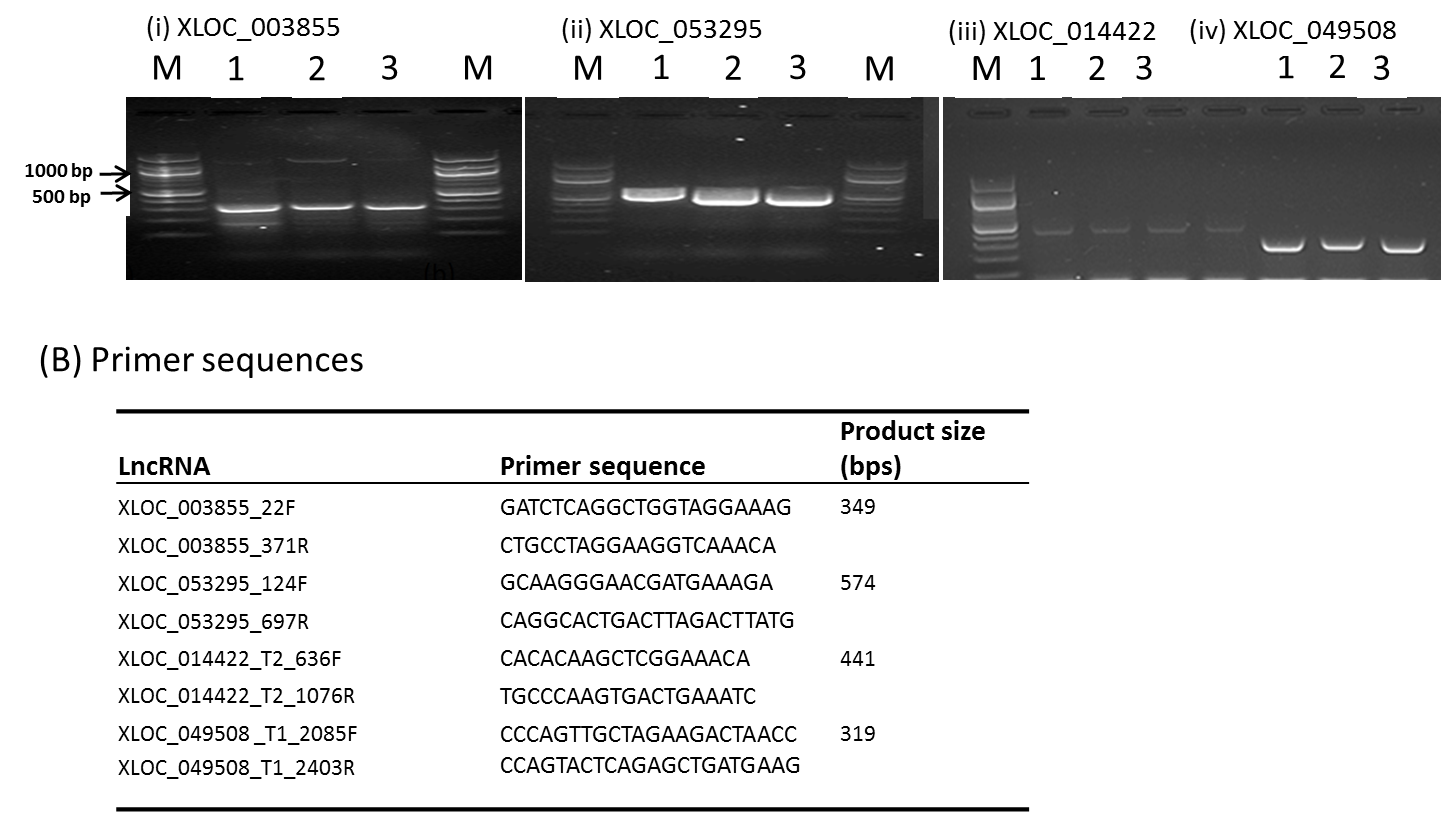

Supplement: Supplementary file 1 [file ijms-19-03610-s001.zip › Supplementary file 6_PCR Products_edit.tif]
